# Enterovirus 71 Outbreak, Brunei

**DOI:** 10.3201/eid1501.080264

**Published:** 2009-01

**Authors:** Sazaly AbuBakar, I-Ching Sam, Jaliha Yusof, Meng Keang Lim, Suzana Misbah, NorAziyah MatRahim, Poh-Sim Hooi

**Affiliations:** University Malaya, Kuala Lumpur, Malaysia (S. AbuBakar, I-C. Sam, S. Misbah, N. MatRahim, P.-S. Hooi); Raja Isteri Pengiran Anak Saleha Hospital, Bandar Seri Begawan, Brunei Darussalam (J. Yusof, M.K. Lim)

**Keywords:** Brunei, outbreak, hand, foot, and mouth disease, human enterovirus 71, dispatch

## Abstract

Enterovirus 71 (EV71) outbreaks occur periodically in the Asia-Pacific region. In 2006, Brunei reported its first major outbreak of EV71 infections, associated with fatalities from neurologic complications. Isolated EV71 strains formed a distinct lineage with low diversity within subgenogroup B5, suggesting recent introduction and rapid spread within Brunei.

Enterovirus 71 (EV71), a member of the family *Picornaviridae* and the genus *Enterovirus*, is a common cause of hand, foot, and mouth disease in children. Infection with this virus is rarely complicated by severe neurologic disease, such as meningitis, brain stem encephalitis, neurogenic pulmonary edema, and acute flaccid paralysis. EV71 was first isolated in 1969 (*1*), and during the subsequent 30 years, outbreaks were reported in the United States, Europe, and Asia (*2*). Since 1997, several major outbreaks with deaths have occurred in the Asia-Pacific region, notably in Sarawak (East Malaysia), Peninsular Malaysia, Taiwan, Australia, Singapore, Japan, and Vietnam (*3*–*10*).

Brunei is situated on the island of Borneo (4°30′N, 114°E) and has a population of ≈370,000. From February through August 2006, Brunei experienced its first reported major outbreak of EV71. More than 1,681 children reportedly were affected, with 3 deaths resulting from severe neurologic disease. We report the virologic findings from this outbreak.

## The Study

During March through October 2006, samples from at least 100 patients from Brunei diagnosed with hand, foot, and mouth disease or herpangina were received at the University Malaya Medical Center, Kuala Lumpur, Malaysia. Samples were inoculated into Vero and A549 cell cultures for virus isolation. EV71 was isolated from 34 patients (including 2 who died of severe neurologic complications), and an additional 7 isolates were obtained from Malaysian patients seen at the University Malaya Medical Center during the outbreak period in Brunei (Table 1). Adenovirus also was isolated from stool or rectal swabs of 4 patients, of whom 2 were coinfected with EV71; none had neurologic disease.

**Table 1:** Enterovirus 71 from Brunei and Malaysia isolated in 2006

Isolate	GenBank accession no.	Subgenogroup	Specimen type	Origin
EV71/BRU/2006/33930	FM201328	B5	Rectal swab	Brunei
EV71/BRU/2006/34095	FM201329	B5	Rectal swab	Brunei
EV71/BRU/2006/34099	FM201330	B5	Rectal swab	Brunei
EV71/BRU/2006/34111	FM201331	B4	Skin swab	Brunei
EV71/BRU/2006/34235	FM201332	B5	Throat swab	Brunei
EV71/BRU/2006/34355	FM201333	B5	Throat swab	Brunei
EV71/BRU/2006/34456	FM201334	B5	Swab*	Brunei
EV71/BRU/2006/34597	FM201335	B5	Stool	Brunei
EV71/BRU/2006/34700	FM201336	B5	Stool	Brunei
EV71/BRU/2006/34701	FM201337	B5	Stool	Brunei
EV71/BRU/2006/35053	FM201338	B5	Rectal swab	Brunei
EV71/BRU/2006/35207	FM201339	B5	Stool	Brunei
EV71/BRU/2006/35245	FM201340	B5	Rectal swab	Brunei
EV71/BRU/2006/35247	FM201341	B5	Rectal swab	Brunei
EV71/BRU/2006/35334	FM201342	B5	Swab*	Brunei
EV71/BRU/2006/35335	FM201343	B5	Blister swab	Brunei
EV71/BRU/2006/35338	FM201344	B5	Swab*	Brunei
EV71/BRU/2006/35341	FM201345	B5	Swab*	Brunei
EV71/BRU/2006/35379	FM201346	B5	Rectal swab	Brunei
EV71/BRU/2006/35479	FM201347	B5	Rectal swab	Brunei
EV71/BRU/2006/35640	FM201348	B5	Rectal swab	Brunei
EV71/BRU/2006/35641	FM201349	B5	Rectal swab	Brunei
EV71/BRU/2006/35643	FM201350	B5	Rectal swab	Brunei
EV71/BRU/2006/35645	FM201351	B5	Rectal swab	Brunei
EV71/BRU/2006/35646	FM201352	B5	Rectal swab	Brunei
EV71/BRU/2006/35649	FM201353	B5	Rectal swab	Brunei
EV71/BRU/2006/35652	FM201354	B5	Rectal swab	Brunei
EV71/BRU/2006/35653	FM201355	B5	Rectal swab	Brunei
EV71/BRU/2006/35728	FM201356	B5	Swab*	Brunei
EV71/BRU/2006/35730	FM201357	B5	Swab*	Brunei
EV71/BRU/2006/35731	FM201358	B5	Swab*	Brunei
EV71/BRU/2006/35732	FM201359	B5	Swab*	Brunei
EV71/BRU/2006/35754	FM201360	B5	Rectal swab	Brunei
EV71/BRU/2006/35755	FM201361	B5	Rectal swab	Brunei
EV71/MY/2006/1764281	FM201321	B5	Stool	Malaysia
EV71/MY/2006/1764283	FM201322	B5	Rectal swab	Malaysia
EV71/MY/2006/1764454	FM201323	B5	Nasopharyngeal swab	Malaysia
EV71/MY/2006/1764589	FM201324	B5	Stool	Malaysia
EV71/MY/2006/1764739	FM201325	B5	Stool	Malaysia
EV71/MY/2006/1765017	FM201326	B5	Stool	Malaysia
EV71/MY/2006/1765058	FM201327	B5	Stool	Malaysia

*Site of swab not known.

Enteroviral RNA was extracted from cell cultures using QIAamp Viral RNA Mini Kit (QIAGEN, Hilden, Germany), and reverse transcription–PCR was performed to amplify the viral capsid protein (VP1) gene at nt positions 31–861. The primers used were VP1F 5′-CAGGCTAGCATGGGAGATAGGGTGGCAGATGTGATCGAGAGC-3′ and VP1R 5′-GGTGGATCCCAAAGGGTAGTAATGGCAGTACGACTAGTGCCGGT-3′. The 831-nt partial VP1 gene fragments were sequenced, and phylogenetic relations of the sequences were examined using selected enterovirus reference strains obtained from GenBank (Table 2). Sequences were aligned and phylogenetic trees were drawn using the neighbor-joining method (Figure), as described (*12*). Maximum-likelihood tree showed similar clustering and is not shown. The prototype coxsackievirus A16 (CoxA16-G10) was used as the outgroup virus for construction of the phylogenetic tree.

**Table 2:** Reference enterovirus 71 sequences used for phylogenetic analysis*

Isolate	GenBank accession no.	Subgenogroup	Origin	Year	Clinical details	Reference
BrCr-CA-70	U22521	A	USA	1970	Encephalitis	(*11*)
S11051-SAR-98	AF376081	C1	Sarawak	1998	HFMD	(*6*)
1M-AUS-12-00	AF376098	C1	Australia	2000	HFMD	(*6*)
2M-AUS-3-99	AF376103	C2	Australia	1999	Myelitis	(*6*)
2644-AUS-95	AF135949	C2	Australia	1995	NA	(*11*)
KOR-EV71-09	AY125973	C3	South Korea	2000	NA	UD
KOR-EV71-10	AY125974	C3	South Korea	2000	NA	UD
F2-CHN-00	AB115491	C4	China	2000	NA	UD
H26-CHN-00	AB115493	C4	China	2000	NA	UD
1091S/VNM/05	AM490143	C5	Vietnam	2005	NA	(*10*)
999T/VNM/05	AM490163	C5	Vietnam	2005	NA	(*10*)
2609-AUS-74	AF135886	B1	Australia	1974	Meningitis	(*11*)
2258-CA-79	AF135880	B1	USA	1979	Tremors	(*11*)
7673-CT-87	AF009535	B2	USA	1987	NA	(*11*)
2222-IA-88	AF009540	B2	USA	1988	Fever	(*11*)
MY104-9-SAR-97	AF376072	B3	Sarawak	1997	Cardiogenic shock	(*6*)
26M-AUS-2-99	AF376101	B3	Australia	1999	HFMD	(*6*)
1067-Yamagata-00	AB213625	B4	Japan	2000	HFMD	(*8*)
2027-SIN-01	AF376111	B4	Singapore	1997	Acute flaccid paralysis	(*6*)
CN04104-SAR-00	AF376067	B4	Sarawak	2000	HFMD	(*6*)
5511-SIN-00	AF376121	B5	Singapore	2000	HFMD	(*6*)
2716-Yamagata-03	AB177816	B5	Japan	2003	HFMD	(*8*)
2419-Yamagata-03	AB213647	B5	Japan	2003	HFMD	(*8*)
S19841-SAR-03	AY258310	B5	Sarawak	2003	NA	UD
SB12869-SAR-03	AY905545	B5	Sarawak	2003	NA	(*3*)

*HFMD, hand, foot, and mouth disease; NA, not available; UD, unpub. data.

**Figure:**
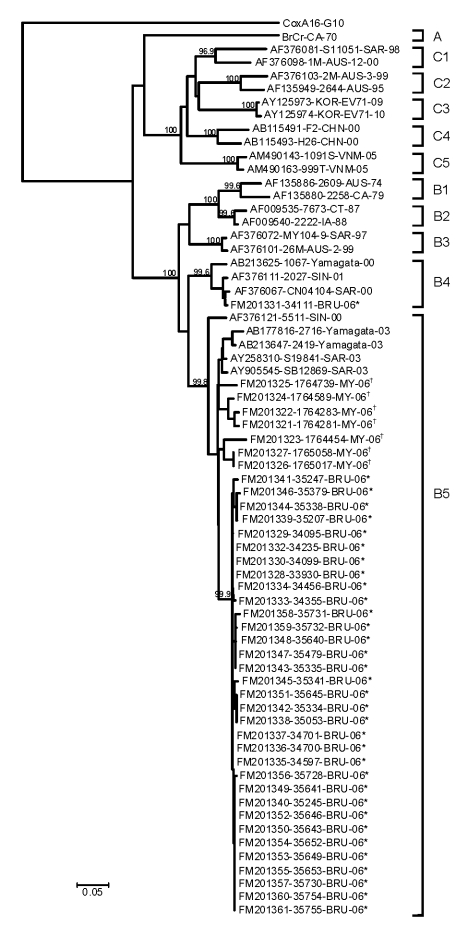
Phylogenetic relationships of enterovirus 71 partial viral protein (VP1) gene sequences. The prototype coxsackievirus A16 (CoxA16-G10) was used as the outgroup virus. The phylogenetic tree shown was constructed by using the neighbor-joining method. Bootstrap values (>95%) are shown as percentages derived from 1,000 samplings at the nodes of the tree. Scale bar denotes number of nucleotide substitutions per site along the branches. Isolates from this study are indicated by * (Brunei) and † (Peninsular Malaysia).

The phylogenetic tree, drawn on the basis of the alignment of the VP1 gene sequences, showed 3 independent genogroups (A, B, and C) with the prototype BrCr strain as the only member of genogroup A (*11*). Within each of genogroups B and C, 5 additional subgenogroups were identified, designated B1–B5 and C1–C5 (*8*,*10*). Although no definitions have been established, generally there is nucleotide variation of ≈16%–20% between genogroups and differences of ≈6%–12% between subgenogroups within each genogroup (*5*,*11*).

All Brunei and Malaysia isolates from 2006 clustered into subgenogroup B5, except for 1 Brunei isolate, which grouped to subgenogroup B4. Nucleotide sequences of the VP1 gene were highly similar (96%–100%) among all strains in subgenogroup B5. All Brunei B5 isolates were clustered in an independent lineage within subgenogroup B5 (99.9% bootstrap support), separate from the established Sarawak and Yamagata isolates from 2003 (*8*). Amino acid sequences were highly conserved among the Brunei B5 isolates, with 99%–100% similarity. No amino acid sequence changes were observed in the 2 isolates from patients who died.

## Conclusions

The different genogroups of EV71 are widely distributed around the world (*2*). The continuing appearance of new EV71 subgenogroups in recent years in the Asia-Pacific region suggests that the virus is continuously evolving (*5*,*8*,*9*). The annual rate of evolution is estimated at 1.35 ×10^–2^ substitutions per nucleotide, similar to poliovirus (*11*). In some countries, outbreaks occur in a cyclical pattern every 3 years, predominantly caused by strains that are distinct from previous outbreaks (*3*,*9*). These strains often have been detected in other countries in the region in years preceding the outbreak. In some EV71 outbreaks, other enteroviruses cocirculate, particularly coxsackievirus A16 or EV71 from a different subgenogroup (*3*,*8*,*10*). On the basis of the samples received in the study, the Brunei 2006 EV71 outbreak was caused by subgenogroup B5 virus. Apart from the single isolate from subgenogroup B4, no other enteroviruses were isolated, although 2 patients also had adenovirus. Occasional EV71 and adenovirus co-infection has been reported (*13*), also without association with severe disease. The low sequence diversity and predominance of the Brunei B5 isolates in this outbreak suggest recent introduction and subsequent rapid spread, without the concurrent spread of other genogroups, subgenogroups, or enteroviruses.

Other than its northern coastline, Brunei is surrounded entirely by the East Malaysian state of Sarawak. In 2006, an outbreak of EV71 affected approximately 14,400 children in Sarawak (*14*). Thus, temporally and geographically, the Brunei and Sarawak outbreaks were related, raising the possibility that the same strains were involved. Sarawak had experienced EV71 outbreaks every 3 years (1997, 2000, and 2003), caused by subgenogroups B3, B4, and B5, respectively (*3*). However, no sequence results from the Sarawak 2006 outbreak are available for comparison. All subgenogroup B5 isolates reported seem to have diverged from an ancestral strain related to strain 5511/SIN/00 (GenBank accession no. AF376121), isolated in Singapore as early as 2000 (*3*). Subsequently, subgenogroup B5 emerged in Japan (*8*) and Sarawak (*3*) in 2003, before appearing in Peninsular Malaysia and Brunei in 2006. The source of the Brunei outbreak remains unclear, and it may not be one of these countries where subgenogroup B5 has already been reported. However, EV71 subgenogroup B5 clearly continues to diverge, and further subgenogroups are likely to arise.

In summary, the first reported major outbreak of EV71 in Brunei was caused by strains from subgenogroup B5 that were distinct from other reported B5 isolates, suggesting a recent introduction from an as-yet-unidentified source. Hence, continued molecular surveillance of EV71 in Asia is required to further our understanding of factors influencing the evolution of the virus and its association with emergence of outbreaks in the region.
